# Spines slow down dendritic chloride diffusion and affect short-term ionic plasticity of GABAergic inhibition

**DOI:** 10.1038/srep23196

**Published:** 2016-03-18

**Authors:** Namrata Mohapatra, Jan Tønnesen, Andreas Vlachos, Thomas Kuner, Thomas Deller, U. Valentin Nägerl, Fidel Santamaria, Peter Jedlicka

**Affiliations:** 1 Institute of Clinical Neuroanatomy, Neuroscience Center, Goethe University Frankfurt, Frankfurt/Main, Germany; 2 Biology Department and Neurosciences Institute, The University of Texas at San Antonio, San Antonio, USA; 3 Interdisciplinary Institute for Neuroscience, CNRS UMR 5297, Bordeaux, France; 4 Institute of Anatomy and Cell Biology, Heidelberg University, Heidelberg, Germany; 5 Interdisciplinary Institute for Neuroscience, University of Bordeaux, France

## Abstract

Cl^−^ plays a crucial role in neuronal function and synaptic inhibition. However, the impact of neuronal morphology on the diffusion and redistribution of intracellular Cl^−^ is not well understood. The role of spines in Cl^−^ diffusion along dendritic trees has not been addressed so far. Because measuring fast and spatially restricted Cl^−^ changes within dendrites is not yet technically possible, we used computational approaches to predict the effects of spines on Cl^−^ dynamics in morphologically complex dendrites. In all morphologies tested, including dendrites imaged by super-resolution STED microscopy in live brain tissue, spines slowed down longitudinal Cl^−^ diffusion along dendrites. This effect was robust and could be observed in both deterministic as well as stochastic simulations. Cl^−^ extrusion altered Cl^−^ diffusion to a much lesser extent than the presence of spines. The spine-dependent slowing of Cl^−^ diffusion affected the amount and spatial spread of changes in the GABA reversal potential thereby altering homosynaptic as well as heterosynaptic short-term ionic plasticity at GABAergic synapses in dendrites. Altogether, our results suggest a fundamental role of dendritic spines in shaping Cl^−^ diffusion, which could be of relevance in the context of pathological conditions where spine densities and neural excitability are perturbed.

Intracellular chloride (Cl^−^) is an important ion involved in the regulation of many cellular properties, from volume and pH regulation to potassium distribution, vesicular trafficking and excitability. In neurons, intracellular Cl^−^ concentration determines the polarity and strength of fast inhibitory transmission mediated by GABA_A_ receptors (GABA_A_Rs), making it crucial to identify the major determinants of intracellular Cl^−^ compartmentalization and dynamics. A large number of studies have focused on detailed mechanisms of transmembrane Cl^−^ transport in nerve cells^1^,^2^,^3^,^4^. However, the impact of complex neuronal morphology on the diffusion and redistribution of intracellular Cl^−^ is poorly understood. Previous studies have shown that the structure of dendrites can alter the diffusion of intracellular molecules like inositol-1,4,5-triphospate or rhodamine dextran, but not calcium^5^,^6^,^7^. The spread of these molecules was strongly slowed down by the presence of dendritic spines^5^,^6^. It is unclear whether spines are able to exert a similar influence on Cl^−^ ions. Previous theoretical studies focused on the electrodiffusion of Na^+^, K^+^ and Cl^−^ within spines^8^,^9^ but did not examine the impact of spines on dendritic Cl^−^ diffusion. This is important because many GABAergic synapses are located directly on dendritic shafts. Hence, we set out to study how dendritic spines affect the spatial spread of intracellular Cl^−^ in the dendritic trunk.

While fluroescence imaging of dendritic Cl^−^ concentration has been successfully achieved^10^,^11^, imaging spatially resolved Cl^−^ changes in dendrites with high temporal resolution has remained a challenge^12^,^13^. Thus, while improved biosensors are in development^13^,^14^, computational modeling is currently the main technique to estimate activity-dependent shifts in dendritic Cl^−^ on micrometer/millisecond scales^4^,^13^, spurring the development of computational models for Cl^−^ diffusion in realistic dendritic morphologies^15^,^16^,^17^. However, dendritic spines have not yet been included in these models, leaving the impact of spines on Cl^−^ diffusion unexplored so far. Therefore, we have used computational models of morphologically complex dendrites with spines to test the effects of spines on dendritic Cl^−^ dynamics and GABA_A_R-mediated inhibition.

Inhibitory synapses exhibit a unique form of plasticity, which depends on short- or long-term shifts in ionic concentration. This phenomenon has been referred to as ionic plasticity^3^,^4^. Fast GABA_A_R-mediated synaptic inhibition relies on the influx of chloride and the efflux of bicarbonate ions^18^,^19^,^20^. Neurons, when exposed to intense inhibitory activity, accumulate intracellular Cl^−^, which substantially modifies the GABAergic reversal potential (E_GABA_)^19^,^20^,^21^,^22^,^23^. Such activity-dependent shifts in Cl^−^ concentration and E_GABA_ underlie short-term ionic plasticity at GABAergic synapses^24^,^25^,^26^. The objective of this study is to understand the effects of dendritic morphology on Cl^−^-dependent short-term ionic plasticity. We have tested the hypothesis that inhibitory synaptic activation results in a local rise in the concentration of dendritic Cl^−^ and the spread of this concentration gradient is not only controlled by transmembrane Cl^−^ transport but strongly affected by the diffusion rate of Cl^−^, which depends on dendritic morphology, i.e. presence of dendritic spines. Our numerical simulations predict that spines influence short-term ionic plasticity by retarding dendritic spread of Cl^−^ ions, yielding a decrease in the apparent diffusion coefficient for Cl^−^.

## Results

### Comparing Cl^−^ diffusion inside smooth and spiny dendrite-like cylinders

**Figure 1:**
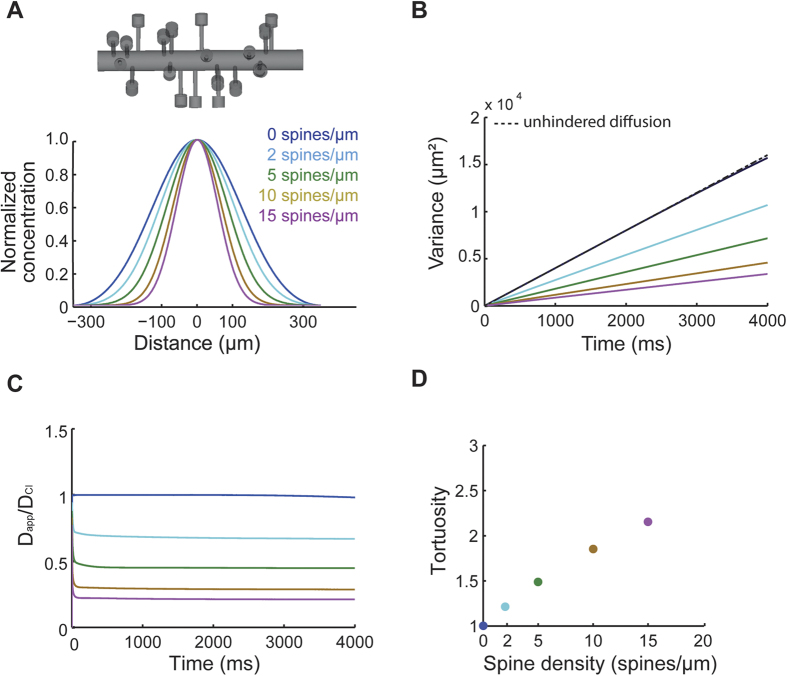
Cl^−^ diffusion is slower in spiny than in smooth dendrite-like cylinders. (**A**) Top: Schematic representation of simulated morphology. Spines were randomly attached to a dendritic cylinder (700 μm length, 1 μm diameter). Bottom: Normalized Cl^−^ concentration profiles at t = 4000 ms in the dendritic cylinder with different spine densities. Cl^−^ diffusion was triggered by an initial increase in Cl^−^ concentration (from 5 to 10 mM at t = 0 ms) within a 1 μm compartment at the center of the dendrite. (**B**) The spatial variance of Cl^−^ concentration as a function of time decreased with increasing spine density. The dotted line denotes the linear time dependence of obstacle-free, unhindered Cl^−^ diffusion. The time dependence of spatial variance indicates a slowdown of diffusion in spiny dendrites. At each time step, the normalized Cl^−^ concentration profiles were used to determine the spatial variance. (**C**) The instantaneous apparent diffusion coefficient (D_app_) was computed from spatial variance and divided by the diffusion coefficient for Cl^−^ (D_Cl_ = 2 um^2^/ms). (**D**) Calculated values of tortuosity as a function of spine density. The tortuosity increased with increasing spine densities.

The major aim of this study was to estimate the effects of dendritic spines on Cl^−^ diffusion in dendrites. For this purpose we first studied the diffusion of Cl^−^ inside long dendrite-like cylinders with different densities of spines using deterministic compartmental diffusion modeling^27^ implemented in NEURON (see Methods). Spines were randomly inserted along an unbranched dendrite with a total length of 700 μm and a diameter of 1 μm. Spines consisted of two concentric cylinders representing head (diameter = 0.6 μm, length = 0.55 μm) and neck (diameter = 0.2 μm, length = 1.25 μm) compartments. The diffusion coefficient of Cl^−^ ions (D_Cl_) has been estimated to be 2 μm^2^/ms^10^. We simulated Cl^−^ diffusion following a focal increase of intracellular Cl^−^ concentration ([Cl^−^]_i_) in the middle of the dendrite (Fig. 1). The spatial spread of Cl^−^ was quantified by determining the variance of the spatial Cl^−^ concentration profile over time. The spatial variance is equivalent to the mean square displacement (MSD) of Cl^−^ molecules along the length of the dendrite (see Methods). The smaller slope of the time course of the spatial variance in spiny dendrites (Fig. 1B) indicated that the diffusion of Cl^−^ was slower than unhindered diffusion. In line with this, spiny dendrites exhibited a decrease in apparent diffusion coefficient of Cl^−^ (D_app_, Fig. 1C) resulting in 20–70% reduction for spine densities of 2–15 spines/μm as compared to the diffusion coefficient in smooth dendrites (D_app_/D_Cl_ ~ 1). To determine whether the diffusion of Cl^−^ was altered by spines, the value of tortuosity was computed from the apparent diffusion coefficient values at the end of the simulation (see Methods). Whereas Cl^−^ diffusion in the smooth dendrite was nearly normal (tortuosity ~1), in spiny dendrites it was slower than normal (tortuosity >1, Fig. 1D). Next, we examined whether the increased volume of the dendrite after adding spines caused this effect. To test this, we performed simulations in smooth dendrites with increased diameters to match the volume of spiny dendrites. The analysis of spatial variance and diffusion permeability showed that changing the diameter, and hence volume, of the dendrite did not alter the spatial spread of Cl^−^ (Supplementary Fig. S1).

**Figure 2:**
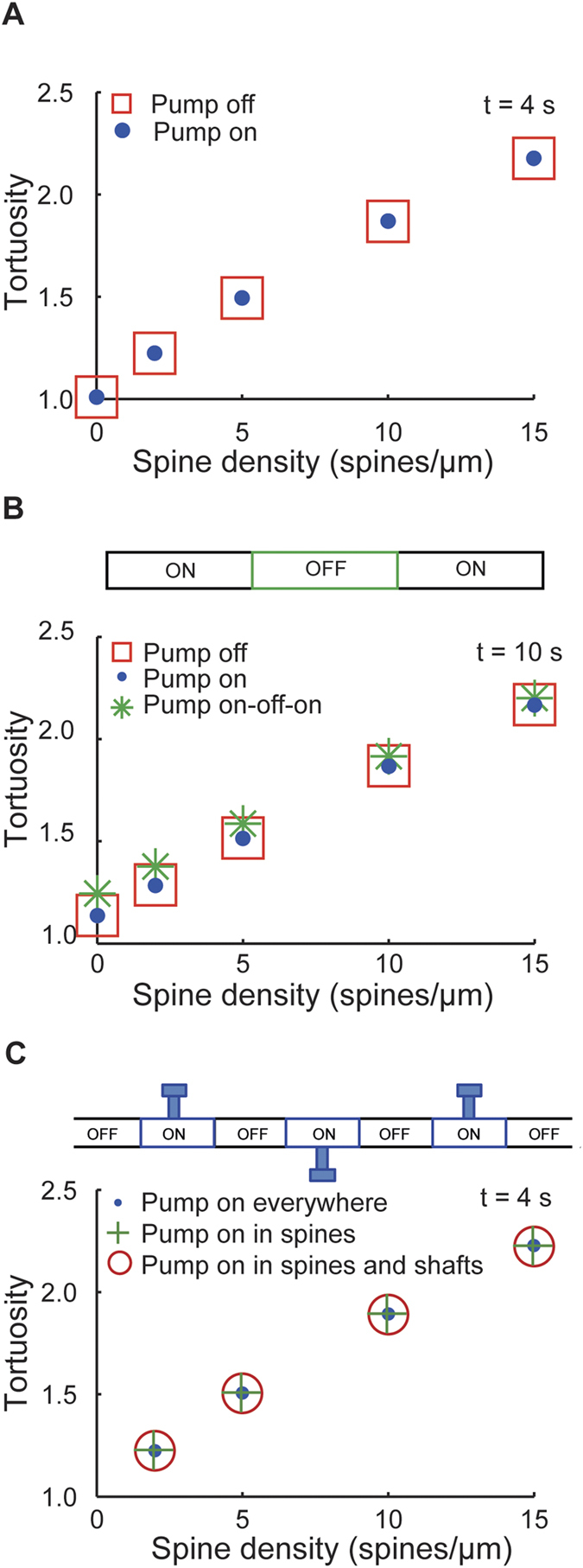
A KCC2-like Cl^−^ extrusion affects the tortuosity of Cl^−^ diffusion to a lesser extent than spines. (**A**) Removal of Cl^−^ ions by a KCC2-like pump was added to simulations from Fig. 1 using exponentially decaying pool model of Cl^−^ concentration. The time constant for Cl^−^ extrusion (τ_Cl_) was 3000 ms. The tortuosity was calculated and compared for 2 different conditions: active Cl^−^ pump (pump on) and inactive Cl^−^ pump (pump off). The presence of active Cl^−^ pump in the dendrite did not change the tortuosity of Cl^−^ diffusion. (**B**) Same simulations as in A but in the presence of nonuniform Cl^−^ extrusion. Top schematic: Active Cl^−^ pump was inserted in two distal thirds of the dendritic shaft. Note a small increase in tortuosity for such nonuniformly distributed Cl^−^ pump (pump on-off-on) as compared to inactive (pump off) or uniformly distributed Cl^−^ pump (pump on). Spines increased tortuosity more than did the nonuniform Cl^−^ pump. (**C**) Same simulations as in A and B but in the presence of active Cl^−^ pump selectively in spines and adjacent dendritic shafts (pump on in spines and shafts, top schematic). For comparison, active Cl^−^ pump was inserted everywhere (pump on) or only in spines (pump on in spines).

Next, we tested whether the effects of spines on diffusional spread remain preserved in the presence of realistic Cl^−^ extrusion. Therefore, we inserted a Cl^−^ pump into the membrane of smooth and spiny dendrites. The pump represented an outward KCC2-like transport mechanism mediating a monoexponential Cl^−^ recovery with a time constant of 3000 ms^22^. The extrusion of Cl^−^ did not lead to a deviation from unhindered diffusion and did not increase the tortuosity (Fig. 2A). However, similarly to previous simulations^5^,^6^,^7^, adding spines affected the diffusion of Cl^−^, resulting in a dramatic decrease of D_app_ and increase of tortuosity. In these simulations, Cl^−^ extrusion was activated homogenously along the entire length of the dendritic membrane. To test, how inhomogenously distributed Cl^−^ pumps influence Cl^−^ diffusion, we repeated the simulations from Fig. 2A in the presence of nonuniform Cl^−^ extrusion (Fig. 2B). Activation of the Cl^−^ pump in two distal thirds of the dendritic shaft caused a small increase in tortuosity as compared to inactive or homogenously activated Cl^−^ pump (see pump on-off-on condition in Fig. 2B). This minor effect was produced by a small reduction in the spatial spread of Cl^−^ owing to decreased Cl^−^ accumulation near sealed ends of the dendrite. Nevertheless, spines affected tortuosity to a much greater extent than the nonuniformly distributed Cl^−^ pump. We obtained similar results when we compared the effects of homogeneously distributed Cl^−^ pump (pump on everywhere) with the Cl^−^ pump distributed only in spines (pump on in spines) or in spines and adjacent dendritic shafts (pump on in spines and shafts). Taken together, simulations in smooth and spiny dendrite-like cylinders revealed that spines can decrease the rate of axial Cl^−^ spreading by 30%.

### Comparing deterministic with stochastic modeling

Diffusion of ions is a stochastic process which might be affected by the interaction of stochastic fluctuations in the movement of ions and space geometry. Therefore, we wondered whether replacing the deterministic model by a stochastic random walk model of Cl^−^ diffusion would lead to similar results. To address this question, we used a voxel-based reaction–diffusion simulator STEPS (STochastic Engine for Pathway Simulation^28^, which implements a spatial version of the Gillespie’s stochastic algorithm. Diffusion of Cl^−^ was simulated as the movement of ions in a volume mesh consisting of tetrahedral voxels^29^. Spines were again modeled as two concentric cylinders representing head (diameter = 0.6 μm, length = 0.55 μm) and neck (diameter = 0.2 μm, length = 1.25 μm), which were attached to a dendritic segment (diameter = 1 μm, length = 100 μm). Chloride ions were released in a 1 μm centered compartment. Since a fraction of Cl^−^ ions entered dendritic spines their lateral movement was slowed down (see Supplementary Movie S7). The quantification of spatial variance in spiny dendrites showed that the diffusion of Cl^−^ was slower than the diffusion occurring in smooth dendrites (Supplementary Fig. S2). The results of stochastic STEPS simulations were similar to those of deterministic simulations, which were run in NEURON (Supplementary Fig. 2). Thus, stochastic modeling supported the observation from deterministic models that spines slow down the longitudinal spread of Cl^−^ ions in spiny dendrites.

### Modeling stochastic Cl^−^ diffusion in realistic reconstructions of dendritic morphology based on STED microscopy

**Figure 3:**
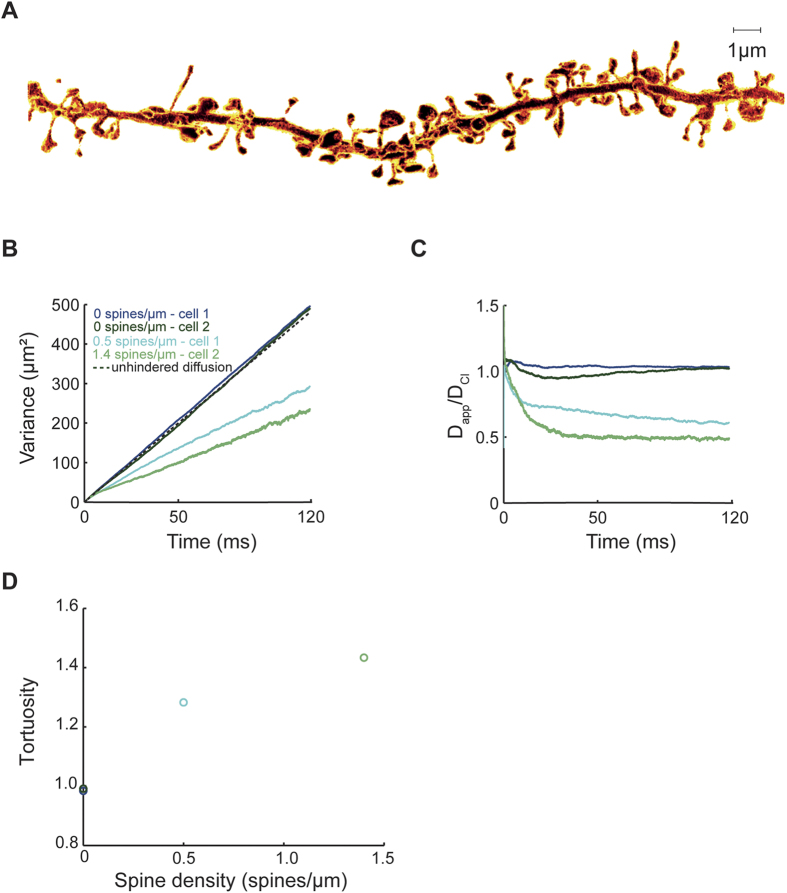
Spines slow down Cl^−^ diffusion in dendrites imaged by live-cell super-resolution STED microscopy. (**A**) STED image of spiny dendritic segment of a CA1 pyramidal cell. (**B**) The spatial variance of Cl^−^ concentration in stochastic simulations of Cl^−^ diffusion in STED-based dendritic morphologies. Note that the spatial variance in spiny dendrites is different from the spatial variance of idealized free (unhindered)Cl^−^ diffusion (dotted line). (**C**) D_app_ was determined from spatial variance (c.f. Fig. 1). The ratio of D_app_ and D_Cl_ of 1 is characteristic for free (unhindered) Cl^−^ diffusion. (**D**) In the presence of spines, the tortuosity was larger than 1 indicating a slower time course of longitudinal dendritic diffusion.

We decided to test whether our predictions will hold under more realistic conditions of stochastic diffusion in real morphologies. Spine parameters such as the neck width may affect the diffusion of molecules in dendrites^5^,^30^. Whereas light microscopy fails to accurately capture the fine morphology of spines, electron microscopy reveals the ultrastructure of spine geometry but only in fixed tissue. However, a recent super-resolution microscopy method based on stimulated emission depletion (STED) enables noninvasive imaging of detailed spine morphology in live neurons^31^,^32^. To test the robustness of the effects of spines on Cl^−^ diffusion we repeated stochastic simulations in morphologies obtained by STED imaging of live spines and dendrites of CA1 neurons (Fig. 3A). The time course of the spatial variance (Fig. 3B) indicated that the diffusion of Cl^−^ was slower in spiny dendrites as compared to their smooth counterparts resulting in a decrease in D_app_ (Fig. 3C). The tortuosity of Cl^−^ diffusion in spiny dendrites was higher than in smooth dendrites (Fig. 3D). Note that, despite lower spine densities, the effect is stronger than in Fig. 1. One reason is that the average head/neck diameter ratio of spines in Fig. 3 is higher than the head/neck diameter ratio of spines in Fig. 1 (3.9 vs. 3, respectively; c.f.^5^). In addition, the spine volume fraction (spine volume/dendrite volume) is also higher in Fig. 3 than in Fig. 1 (c.f. Table S1), illustrating that diffusional retardation by spines is more pronounced in thinner dendrites. In sum, these simulations confirmed and extended our previous observations that spines significantly alter the diffusion geometry of dendrites and increase the tortuosity of Cl^−^ movement by introducing a diffusion delay along the dendrite.

### Predicting Cl^−^ diffusion in the dendrites of reconstructed neurons with full morphology

**Figure 4:**
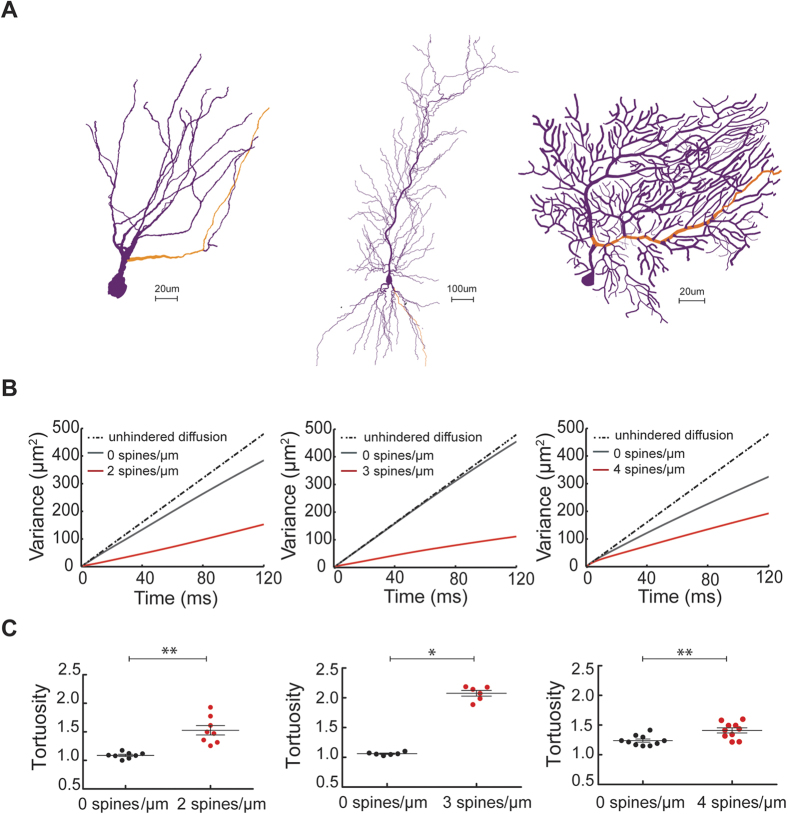
Spines slow down Cl^−^ diffusion in reconstructed dendritic trees with realistic branching. (**A**) Example of a branched dendritic architecture in a dentate granule cell, a CA1 pyramidal cell and a cerebellar Purkinje cell. The site of the initial increase of Cl^−^ concentration was located in the middle of the orange path. (**B**) The plot of the spatial variance of Cl^−^ spread versus time shows that the variance decreased in dendrites covered with spines. (**C**) Calculated tortuosity values indicate a substantial influence of spines on Cl^−^ diffusion in dendritic trees of granule, pyramidal and Purkinje cells.

The preceding computational experiments were performed in unbranched dendritic structures, i.e. parts of dendritic segments. To examine whether the predicted spine effects are also seen when tested in dendritic trees with realistic branching patterns, we ran similar simulations using the reconstructed dendritic morphologies of entire neurons. To this end, we employed NEURON simulations to predict the diffusion of Cl^−^ in three different cell types (Fig. 4A): hippocampal dentate granule cells, hippocampal CA1 pyramidal cells and cerebellar Purkinje cells. After a focal increase in [Cl^−^]_i_ in the middle of a randomly chosen dendrite, we tracked the spread of Cl^−^ and the corresponding spatial variance of Cl^−^ concentration along that dendrite. In this way we compared Cl^−^ diffusion within smooth dendritic trees to the diffusion within dendritic trees with realistic densities of spines (2, 3 and 4 spines/μm in granule, pyramidal and Purkinje cells, respectively). The geometrical parameters of spines were based on experimentally determined values for the diameter and the length of the spine head and neck^5^,^33^. In each of the three cell groups, the insertion of spines resulted in lower slopes for time dependence of spatial variance (Fig. 4B) and in higher values of tortuosity (Fig. 4C), indicating that the presence of spines dramatically slowed down the diffusion of Cl^−^ within dendrites of all three types of neurons. The presence of branches also affected the spatial variance and the tortuosity as evidenced by the results of simulations in smooth dendritic trees, which were slightly different from results in unbranched dendritic cylinders (c.f. Figs 1 and 2 with Fig. 4B,C).

### Predicting the effects of dendritic spines on short-term ionic plasticity

**Figure 5:**
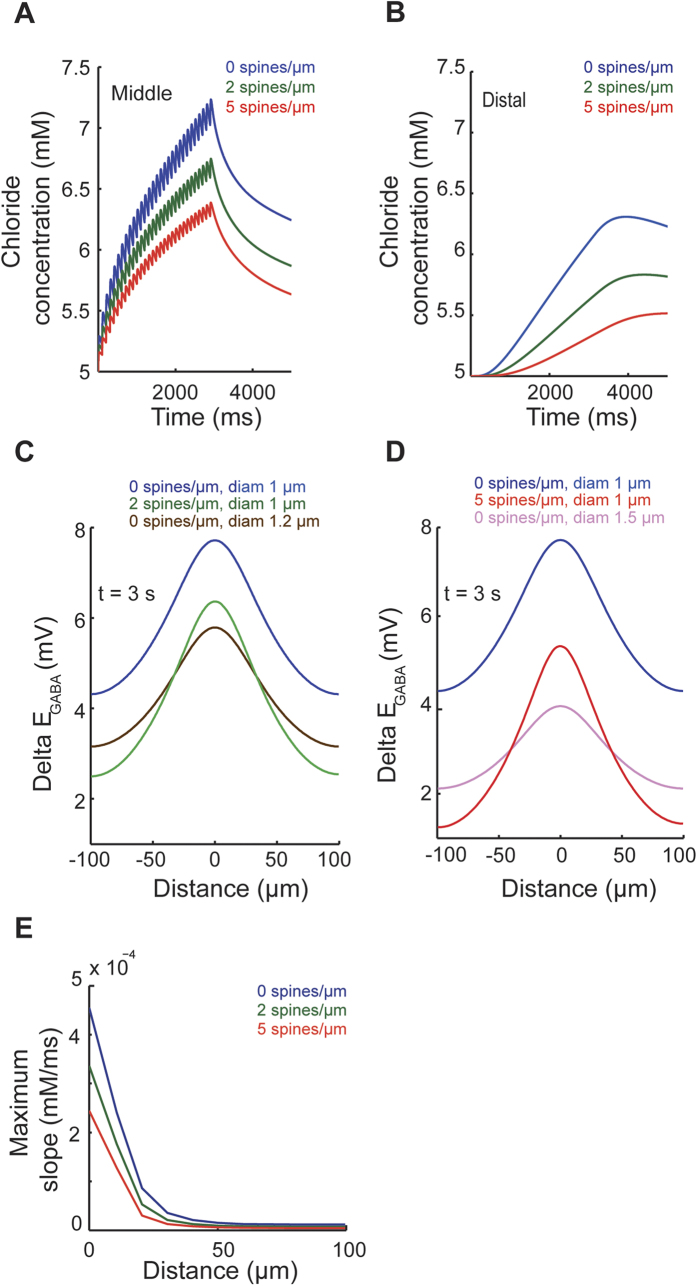
Spines affect short-term ionic plasticity following repetitive activation of GABAergic dendritic inputs. GABAergic synaptic inputs were inserted into the central part of a dendritic cylinder (diameter 1 μm, length 200 μm) and activated (11 dendritic shaft synapses, density 0.5/μm, rise time 0.5 ms, decay time 6 ms, conductance 1 nS). Activity-dependent Cl^−^ accumulation and E_GABA_ shift were recorded as measures of short-term ionic plasticity. (**A**,**B**) Changes in Cl^−^ concentration in the middle (**A**) and at the distal end (**B**) of the dendrite brought about by synchronous repetitive activation (10 Hz, 30 pulses) of GABAergic inputs. (**C**,**D**) Spatial profile of Cl^−^ accumulation and E_GABA_ shift across the length of the dendrite recorded at the end of GABAergic activity (t = 3000 ms). Note that, in the presence of spines, Cl^−^ accumulation and E_GABA_ shift was decreased both in the central as well as in the distal parts of the dendrite indicating a reduction in homosynaptic as well as heterosynaptic ionic plasticity. Similar effects were observed in simulations of stochastic GABAergic activity (Supplementary Fig. 3) and in reconstructed morphologies with realistic branching (Supplementary Fig. 4). In contrast, following adjustments of the total dendrite volume (using a compensatory increase in dendrite diameters of smooth dendrites), spines decrease heterosynaptic spread of ionic plasticity to distal dendritic areas but increase the local, homosynaptic ionic plasticity. The dendrite with 2 spines/μm (or 5 spines/μm) and 1 μm diameter has the same volume as the one with 0 spines/μm and 1.2 μm diameter (or 1.5 μm diameter). (**E**) Maximum slope of Cl^−^ transients across different locations along the dendrite for the initial 3000 ms.

The analysis of the spatial variance of Cl^−^ concentration clearly showed that dendritic spines are capable of limiting the spatial spread of Cl^−^. Next, we examined how this might affect E_GABA_ and inhibitory synaptic transmission. First, we studied Cl^−^ and E_GABA_ dynamics following activation of GABA_A_ synapses in a dendritic cylinder of 200 μm length and 1 μm diameter. The GABA_A_ synapses were equipped with realistic kinetics (rise time = 0.5 ms, decay time = 6 ms, peak conductance = 1 nS) and placed on a 22 μm long section centered at 100 μm from the end of dendrite at a density of 0.5/μm (n = 11). These 11 GABA_A_ inputs were activated synchronously at 10 Hz frequency for 3000 ms. The active Cl^−^ pump was also included. Spines were randomly attached to the dendrite and the spine parameters were identical to those used in the Fig. 1. Figure 5A,B illustrates the temporal changes of Cl^−^ accumulation and E_GABA_ at a middle (110 μm) and a distal (190 μm) location along the dendrite with respect to the GABA_A_ synapse location. Repetitive activation of GABA_A_ inputs led to a stronger accumulation of Cl^−^ in the smooth dendrite (diameter 1 μm) as compared to the spiny dendrites (2 and 5 spines/μm, diameter 1 μm). This was the case along the entire dendritic length, since, at the end of synaptic activation (t = 3000 ms), higher [Cl^−^]_i_ was detected at each dendritic location in the smooth dendrite (Fig. 5C,D). The results were similar for simulations of GABAergic activity in reconstructed morphologies (Supplementary Fig. S3) as well as for a stochastic activation of GABA_A_ inputs by Poisson spike trains (Supplementary Fig. S4). Interestingly, simulations in Fig. 5A,B revealed that the transient Cl^−^ changes (arising due to repetitive synaptic Cl^−^ influx) were low-pass filtered along the dendrite. To quantify the spatial extension of this low-pass filtering effect, we plotted the maximum slope of Cl^−^ transients at different locations along the dendrite (Fig. 5E). This analysis showed that fast Cl^−^ transients were smoothed out at ~50 μm from the dendritic entry point.

In previous simulations, adding spines increased the volume of the dendrite. Although the spatial spread of Cl^−^ in smooth dendrites, as quantified by the spatial variance of relative Cl^−^ changes, is not affected by the total volume of the dendrite (c.f. Supplementary Fig. S1), this is not the case for absolute changes in [Cl^−^]_i_ and E_GABA_. The reason is that increasing the volume decreases the accumulation of Cl^−^. Therefore, we tested how dendritic spines influence Cl^−^ concentration and E_GABA_ after removing the volume effect. To this end, we increased the diameter of the smooth dendrite and performed similar simulations as before (Fig. 5A,B). When compared to volume-compensated dendrites (diameter 1.2 or 1.5 μm), spines (with densities 2 or 5 spines/μm) attenuated Cl^−^ accumulation and the resulting decrease in E_GABA_ in distal dendritic regions, but enhanced it in the central part of the dendrite where GABAergic inputs were activated (Fig. 5C,D). Thus, after removing the volume effect of spines, the spine-mediated decrease in the spatial spread of Cl^−^ becomes dominant. Therefore, whereas Cl^−^ accumulates more at the synaptic site, the nearby dendrite sees a smaller change in Cl^−^ concentration and E_GABA_ (compare the green curve to the brown one in Fig. 5C and the red curve to the violet one in Fig. 5D). This indicates that spines attenuate heterosynaptic ionic plasticity but augment local, homosynaptic ionic plasticity relative to smooth dendrites.

### Predicting the effects of dendritic spines on neuronal excitability

**Figure 6:**
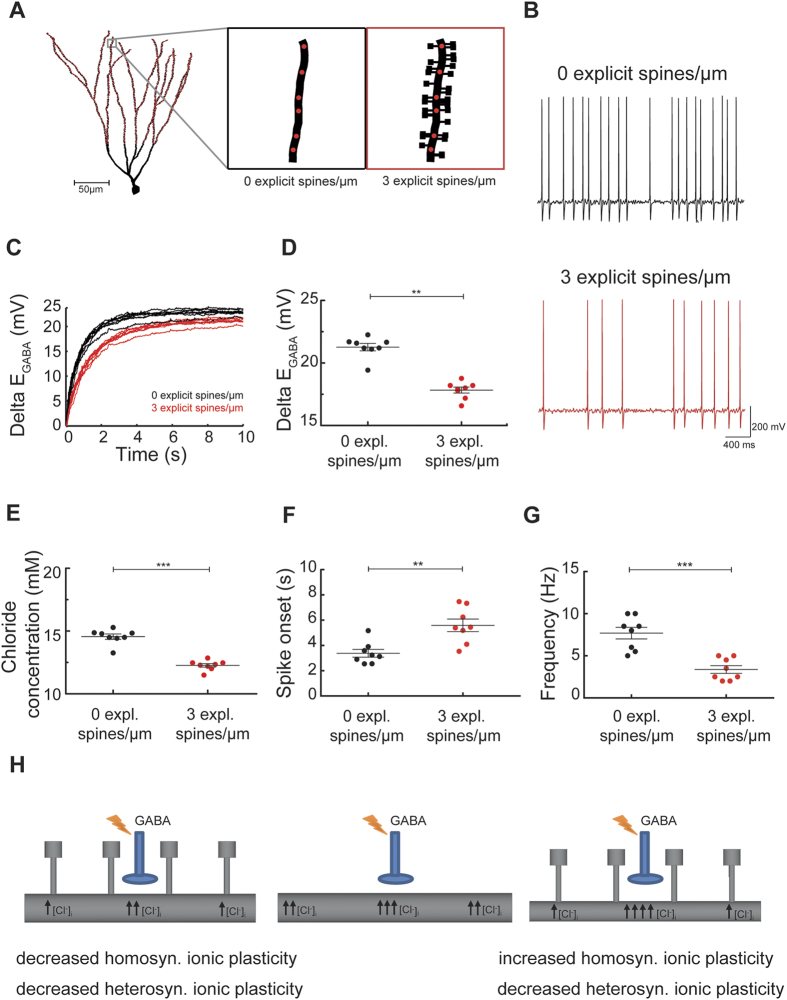
Spines reduce activity-dependent Cl^−^ accumulation, GABAergic hyperpolarization-depolarization switch and firing in dentate granule cells. (**A**) Example of three-dimensional morphology of a dentate granule cell, which was subjected to intense activation of dendritic GABAergic synapses. GABA_A_ synapses (1.5 nS) were placed on the dendritic shaft in the distal two thirds of the dendritic tree (in the outer and middle molecular layer) and their position is indicated by red circles. (**B**) Somatic voltage trace from a granule cell with explicit (red) and implicit spines (black). Note that activation of GABAergic synapses (5 Hz) induced Cl^−^ accumulation and a switch from inhibition to excitation. Driven by GABAergic excitation, the cell with explicit spines was firing with lower spike frequency than the cell with implicit spines. (**C**) Temporal profile of dendritic E_GABA_ change for granule cells (n = 8) with explicit (red) and implicit spines (black). (**D**,**E**) Comparison of dendritic E_GABA_ change and chloride concentration (200 μm from soma, 3 s) in granule cells with explicit and implicit spines, respectively. (**F**) Time to first spike (spike onset) was delayed in the presence of explicit spines. (**G**) The presence of explicit spines led to a significant decrease in the firing frequency computed for the last 2 seconds of dentate granule cells. (**H**) Adding spines to a smooth dendrite decreases both homosynaptic as well as heterosynaptic ionic plasticity at GABAergic inputs (left) as compared to a smooth dendrite with identical diameter (middle). Spiny dendrite (right) with a smaller diameter and a volume identical to the smooth dendrite (middle) displays enhanced homosynaptic but reduced heterosynaptic ionic plasticity.

Finally, we tested how spine-dependent alterations of short-term ionic plasticity might affect neuronal excitability. To this end, we simulated a reduction in E_GABA_ in active models of granule cells with realistic spine densities. We compared E_GABA_ changes and excitability in morphologies (n = 8) with and without explicit spines (i.e. modeled as cylindrical compartments). Passive properties (input resistance, membrane time constant) were kept identical in the two groups of modeled neurons. This was achieved by a precise compensatory insertion of implicit spines in cells, which did not contain explicit spines. Implicit spines were modeled by scaling membrane resistance and capacitance. We inserted and activated GABAergic synapses in the two distal thirds of hippocampal granule cell dendrites, which correspond anatomically to the outer and middle molecular layer (Fig. 6A). As observed in our previous work^15^ stochastic background activity of GABAergic synapses induced strong Cl^−^ accumulation and depolarization of E_GABA_. As a result GABAergic synapses became excitatory and granule cells started firing action potentials (Fig. 6B). However, neurons with explicit spines exhibited a significantly slower and smaller change of E_GABA,_ and [Cl^−^]_i_ (Fig. 6C–E) which led to increased delay of firing and lower firing rates (Fig. 6F,G). These simulations indicate that spines are able to attenuate activity-dependent reductions in E_GABA_ and associated increase in neuronal excitability.

## Discussion

The main finding of our modeling study is that spines retard longitudinal diffusion of Cl^−^ in neuronal dendrites. We found that locally increased [Cl^−^]_i_ spread more rapidly throughout smooth dendrites than spiny dendrites resulting in D_app_ smaller than D_Cl_. The reduced D_app_ reflected an increase in tortuosity that was proportional to spine density. This dependence was present in diverse realistic morphologies, including reconstructions based on STED images of live neurons, indicating that spines are able to hinder Cl^−^ diffusion regardless of neuron type. Importantly, the diffusion coefficient and the dendritic structure were the only constraints needed to fully characterize these simulations. The second major prediction of our modeling is that Cl^−^ diffusion is much less influenced by the presence of KCC2-like Cl^−^ extrusion mechanisms than by the presence of spines. This is due to the fact that Cl^−^ diffusion is fast and redistributes Cl^−^ on a time scale of milliseconds whereas Cl^−^ extrusion is slow and operates on a time scale of seconds. Finally, the third prediction of our work is that, by limiting the spatial spread of Cl^−^, spines affect short-term ionic plasticity of GABAergic synapses and neuronal excitability.

### Spines delay longitudinal spread of Cl^−^ along dendrites

Previous theoretical and experimental analyses showed that spines hinder the diffusion of intracellular inositol-1,4,5-triphospate and rhodamine dextran^5^,^6^. In contrast, the diffusion of calcium is not appreciably affected by dendritic spines. Rather calcium diffusion is strongly curtailed by the action of calcium buffers, which quickly and effectively reduce the free calcium concentration^5^,^7^. Whereas the spread of free calcium is limited to a few microns in dendrites, Cl^−^ ions can spread over tens of micrometers in the absence of fast buffering mechanisms^10^,^15^,^16^. Our simulations indicate that the actual size of the dendritic region covered by spreading Cl^−^ ions will strongly depend on the presence of spines in that region. We have observed that spines had the largest contribution to the decrease of the apparent diffusion coefficient for Cl^−^. However, there was also a considerable contribution from dendritic branching and only minimal contribution from Cl^−^ extrusion. Previous work showed that the presence of dendritic branching has a relatively small effect on the diffusion of inositol-1,4,5-triphospate and rhodamine dextran, which diffuse slowly by comparison^5^. In the case of highly diffusible Cl^−^ ions, the branching of the dendrite had a larger effect. In contrast to inositol-1,4,5-triphospate and rhodamine dextran^5^, the influence of branching on Cl^−^ diffusion was significant and depended on the level of dendritic arborization but it did not abolish the influence of spines. Thus, we expect the effects of spines on Cl^−^ diffusion to be present in all cell types with spiny dendrites. This prediction is supported by the highly robust simulation results obtained in three different settings: artificial dendritic cylinders, realistic dendritic segments imaged by superresolution STED microscopy as well as 3D-reconstructed branched morphologies of dentate granule, CA1 pyramidal and cerebellar Purkinje cells. Of interest, although we were focusing on Cl^−^ it is likely that our results can be extended to the dendritic diffusion of other ion species such as Na^+^. Simulations of Na^+^ diffusion might help better understand recent data from multi-photon Na^+^ imaging in spiny dendrites^34^.

What is the mechanism underlying the increase in the tortuosity of dendritic Cl^−^ diffusion? A key factor is the increase in the diffusion path length in spiny dendrites. Insertion of dendritic spines creates additional space in which Cl^−^ ions get delayed. The effect of spines is similar to placing transient buffers along the dendrite or to adding dead-end pockets perpendicularly to the longitudinal axis of the diffusion flow^35^. Cl^−^ ions which enter the dead-end space of spines will spend extra time dwelling inside spine before they return to the dendrite and continue diffusing in the longitudinal direction. Cl^−^ ions which were hiding in spines and return cause a slower relaxation of the Cl^−^ gradient along the dendritic shaft. Of note, the limited speed of Cl^−^ diffusion in spiny dendrites, as quantified by increased tortuosity, does not imply that the diffusion of Cl^−^ is hindered at the submicrometer scale. The diffusion remains free at the molecular scale but appears retarded at the micrometer scale along the dendritic shaft.

In principle, the diffusion of Cl^−^ through the cytoplasm of dendrites can be affected by the following three factors: (A) the 3D-geometry of the dendrite and its intracellular space, (B) binding and transport of Cl^−^ by proteins in the cell membrane or in the membrane of intracellular organelles, and (C) the presence of immobile electric charges. These factors are similar to factors, which are thought to influence the diffusion of charged molecules in the extracellular space^36^. Our work predicts that the factor (A) is a key determinant of longitudinal cytoplasmic movement of Cl^−^ ions in neuronal dendrites. An important question that remains to be addressed is whether intradendritic organelles such as mitochondria or endoplasmic reticulum^37^ may accentuate the impact of factor (A) or (B) by restricting the intracellular space available for Cl^−^ diffusion or by removing Cl^−^ ions from the cytoplasm, respectively. In these two ways, intracellular organelles might contribute to slowing down longitudinal Cl^−^ spread. Moreover, intracellular organelles might cause anisotropy of diffusion, giving rise to different diffusion speed along the dendrite as compared to across it. More work is needed to determine how a difference in the relative speed of Cl^−^ diffusion in spines vs. shaft might affect the contribution of spines. Of note, in our simulations spines induce a clear tortuosity effect even for slower values of the free Cl^−^ diffusion coefficient. Regarding factor (C), a recent study suggests that [Cl^−^]_i_ is strongly influenced by the presence of immobile negative charges^38^, however this conclusion has been called into question by others^39^,^40^. Future experiments and simulations in detailed 3D models implementing Cl^−^ electrodiffusion^16^ will help clarify the impact of fixed negative charges on Cl^−^ homeostasis^41^. Since we focused on Cl^−^ dynamics in dendrites but not on detailed Cl^−^ diffusion within spines, we did not use an electrodiffusion equation^8^,^9^. However, a potential limitation of our model is that negative charges at the inner surface of the membrane at the spine neck may limit the movement of Cl^−^ into and out of spines thereby modulating the tortuosity effect.

### Spines affect Cl^−^-dependent short-term ionic plasticity at GABAergic synapses

It is well established that intense activation of GABA_A_ synapses leads to short-term ionic plasticity of GABAergic synapses due to highly localized influx of Cl^−^, outflow of bicarbonate and subsequent changes in [Cl^−^]_i_^19^,^20^,^21^,^22^,^23^,^24^,^25^,^26^. Our simulations indicate that spines can alter short-term ionic plasticity in two ways: first, by limiting the spatial spread of Cl^−^ and corresponding E_GABA_ changes and, second, by increasing the volume of the dendritic compartment into which Cl^−^ flows and accumulates. Simulations allowed us to discriminate between these two effects. The quantification of diffusion permeability and tortuosity indicates that in smooth dendrites the spatial spread of Cl^−^ is independent of dendrite volume. The reason for this is that volume averaging makes the apparent diffusion coefficient dependent only on the duration of diffusion and the average displacement of Cl^−^ ions. However, absolute Cl^−^ concentration and its spatial and temporal changes depend strongly on the volume of the dendrite^15^,^22^. Here, we have shown that the enlargement of the dendritic volume by spines is sufficient to decrease Cl^−^ accumulation and the subsequent E_GABA_ shift evoked by GABAergic synaptic activity. This volume effect of spines reduces focal ionic plasticity (i.e. E_GABA_ changes close to the site of synaptic Cl^−^ influx) but the diffusion-limiting effect of spines also reduces ionic plasticity in adjacent dendritic areas. In other words, spines limit both homosynaptic as well as heterosynaptic ionic plasticity at GABAergic inputs (Fig. 6H, left). On the other hand, after removing the volume effect by a compensatory increase in dendrite diameter, a different situation is observed. In this case, the presence of spines again hinders the spread of Cl^−^ and corresponding E_GABA_ change to adjacent areas, but at the same time it enhances the focal accumulation of Cl^−^ and focal E_GABA_ shift. Thus, if a dendrite counteracted the increase in its volume (caused by adding spines or enlarging them) by decreasing its diameter, spines would limit heterosynaptic but boost homosynaptic ionic plasticity (Fig. 6H, right). In experiments, LTP induction sometimes leads to an increase in spine size or density^31^ which is not accompanied by changes in the diameter of the dendrite. We predict that such morphological changes may locally reduce ionic plasticity in dendritic segments. This mechanism could contribute to the maintenance of effective inhibition in potentiated dendritic branches, thereby upholding the excitation/inhibition balance within dendrites which exhibit clustered structural synaptic plasticity^42^.

### Experimental tests and clinical relevance

Although currently available Cl^−^ indicators have limited spatial and temporal resolution, new optical methods of Cl^−^ measurements are being developed^12^,^13^. Thus, future studies - which depend, however, on further advances in imaging and electrophysiology – could test our predictions directly by imaging and measuring Cl^−^ dynamics in smooth and spiny dendrites. Two-photon photolysis of GABA could be used to map the spatial distribution of GABAergic synapses and to quantify the spread of activity-dependent GABAergic Cl^−^ changes in smooth and spiny dendrites.

The clarification of mechanisms regulating Cl^−^ dynamics and ionic plasticity is important for a deeper understanding of neurological diseases associated with hyperexcitability. It is well known that the disruption of Cl^−^ homeostasis and the collapse of Cl^−^ gradients contribute to various pathological conditions^1^ such as epilepsy^43^, pain, anxiety^44^, stress^45^ and schizophrenia^46^. Our simulations suggest that spines might serve as a protection mechanism for limiting the collapse of intradendritic Cl^−^ gradients evoked by intense GABAergic activity. In this way, spines may help preserve the stability of dendritic GABAergic inhibition. Indeed, E_GABA_ is usually more stable in principal neurons with spiny dendrites than in interneurons with smooth dendrites^47^. The collapse of Cl^−^ gradients in interneurons would not lead to pathological hyperexcitability since it would reduce their inhibition, enhance their excitability and thereby promote network inhibition, which would counteract epileptic tendencies. This might be one of the reasons why most GABAergic interneurons have smooth dendrites. Another hint for a close association between spines and Cl^−^ homeostasis is the presence of the Cl^−^ pump KCC2 in the vicinity of spines^48^. KCC2, via its effects on spinogenesis^49^ and Cl^−^ extrusion^4^, might provide double protection for the stability of Cl^−^-dependent inhibition.

## Methods

### Compartmental modeling of Cl^−^ diffusion and extrusion

For deterministic simulations, standard compartmental diffusion modeling based on Fick’s diffusion laws was used^27^. The model did not include electrodiffusion effects. To characterize the diffusion of Cl^−^ in the presence of spines, we adopted our previously published model^15^. Longitudinal Cl^−^ diffusion along dendrites was modeled as the exchange of Cl^−^ between adjacent compartments. For radial diffusion, the volume was discretized into a series of 4 concentric shells around a cylindrical core^27^ and Cl^−^ was allowed to flow between adjacent shells^50^. The free diffusion coefficient of Cl^−^ (D_Cl_) inside neurons was set to 2 μm^2^/ms^10^ which is close to that in free aqueous solutions. However, the effects of spines were present also at lower values of D_Cl_.

Cl^−^ extrusion: In deterministic compartmental simulations, a pump mechanism for transmembrane Cl^−^ transport was included. Cl^−^ extrusion was modeled as exponential recovery of [Cl^−^]_i_ to its resting level [Cl^−^]_i_^rest^ with a decay time constant τ_Cl_ of 3000 ms^22^:


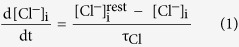


The decay approximates an outward KCC2-like Cl^−^ transport mechanism with first order kinetics^22^.

Cl^−^ influx via GABAergic synapses: The contribution of GABAergic chloride currents to the Cl^−^ concentration inside the cellular compartment was calculated as:


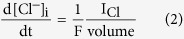


In each compartment, membrane potential was affected by activity-dependent (E_Cl_) and activity-independent (E_rest_, E_HCO3_) reversal potentials. GABA_A_ synapses were simulated as a postsynaptic parallel Cl^−^ and HCO_3_^−^ conductance with exponential rise and exponential decay^15^:


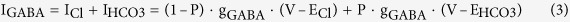


where P is a fractional ionic conductance that was used to split the GABA_A_ conductance (g_GABA_) into Cl^−^ and HCO_3_^−^ conductance. E_Cl_ and E_HCO3_ were calculated from Nernst equation. To calculate g_GABA_, we implemented a double-exponential function with rise time (tau1), decay time constant (tau2) and a peak conductance (g_max,_ see below) by adapting an Exp2Syn synapse in NEURON (see gabaA_Cl.mod file on ModelDB, accession number 148253). All GABAergic synapses were targeting dendritic shafts^51^, no GABAergic spine synapses were included.

Compartmental simulations were run in the simulation environment NEURON ( www.neuron.yale.edu). The script for GABAergic synaptic and ionic mechanisms was written in the model description language NMODL^50^. Parameters used in our simulations were as follows: [Cl^−^]_i_ = 5 mM, [Cl^−^]_o_ = 133.5 mM, [HCO_3_^−^]_i_ = 16 mM, [HCO_3_^−^]_o_ = 26 mM, E_GABA_ = −68.63 mV, temperature 35 °C, P = 0.25, relative conductance of HCO_3_^−^ vs. Cl^−^ P/(1 – P) = 0.25/0.75 = 0.33, which was in line with the published relative permeability values^52^. D_Cl_ = 2 μm^2^/ms; g_GABA_ of dendritic synapses: tau_1_ = 0.5 ms, tau_2_ = 6 ms, g_max_ = 0.5 nS^53^, V_rest_ = −70 mV, passive properties were taken from^54^.

### Stochastic modeling of Cl^−^ diffusion

For stochastic simulations, we subdivided the 3D space of a dendrite into irregular tetrahedral compartments using the volume discretization software CUBIT ( www.cubit.sandia.gov). The diffusion of Cl^−^ was simulated as 3D random transfer of Cl^−^ particles from one tetrahedron to another using spatial Gillespie’s Stochastic Simulation Algorithm. The algorithm was implemented in STEPS (STochastic Engine for Pathway Simulation) which is a software for exact simulations of reaction-diffusion systems. The tetrahedral mesh was imported into STEPS. The diffusion coefficient (D_Cl_ = 2 μm^2^/ms) was the only parameter needed to fully characterize the simulation.

### Diffusion permeability and tortuosity

In all simulations, the apparent diffusion coefficient (D_app_) as a function of time was computed using the equation:


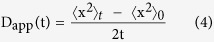


where t is time and x^2^ is the spatial variance of the Cl^−^ concentration profile at each time point which is equivalent to the mean square displacement (MSD) of diffusing Cl^−^ ions from their initial position. <x^2^>_0_ is the spatial variance of the initial condition. The variance was calculated as


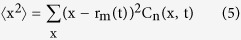


where C_n_(x, t) is normalized spatial profile of Cl^−^ concentration, r_m_(t) is the mean (centroid) at each time point^5^,^6^. To quantify the average hindrance of Cl^−^ diffusion in spiny dendrites relative to diffusion-free smooth dendrites, the tortuosity (λ) was calculated from the ratio of the free diffusion coefficient D_Cl_ and the apparent diffusion coefficient D_app_^36^:


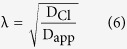


Whereas for obstacle-free (unhindered) diffusion the value of λ is 1, for completely blocked diffusion the value of λ is infinity. Analysis of simulation results was done using Matlab routines.

### Morphology of dendrites, neurons and spines

Simulations of Cl^−^ diffusion were performed in different dendritic morphologies with randomly attached dendritic spines of different shapes. First, for initial analyses, spines of increasing densities were added perpendicularly to a smooth cylinder of length 700 μm and diameter 1 μm. Each spine was modeled as a concatenation of two concentric cylindrical compartments. One cylinder represented the spine neck and the other represented the spine head. The following spine parameters were used (Supplementary Table S5): head diameter 0.6 μm, head length 0.55 μm, neck diameter 0.2 μm and neck length 1.25 μm^5^. Cl^−^ diffusion was triggered by focal increase of Cl^−^ in the middle section of the dendrite or by activating GABA_A_ synapses (see above). Next, Cl^−^ dynamics was analyzed in morphologies constructed in CUBIT using STED images of dendrites and spines from cultured hippocampal slices (see below). The morphologies were imported into the STEPS package and contained experimentally determined dendritic diameters, spine locations as well as widths and lengths of spine necks and heads. To obtain dendritic geometries of sufficient length, identical copies of imaged segments (n = 9 for cell1, n = 5 for cell 2) were concatenated and used in stochastic simulations. Finally, large scale simulations of Cl^−^ diffusion were run in realistic whole-cell morphologies which were reconstructed from light-microscopy images: dentate granule cells (8 cells from^54^, ModelDB – Accession Number: 95960), CA1 pyramidal cells (3 cells from^55^, ModelDB – Accession Number: 64170; 3 cells from^56^) and cerebellar Purkinje cells (10 cells with NeuroMorpho.ID: NMO_00863; NMO_00862; NMO_00864; NMO_00865; NMO_10069; NMO_10070; NMO_10071; NMO_10072; NMO_10073; NMO_10074). Realistic spines were randomly attached to the surface of these neurons using realistic cell type specific parameters (Supplemenary Table S6).

### STED microscopy of dendrites and spines

STED images of live dendritic spines were obtained in cultured hippocampal slices isolated from Thy1-YFP mice (Jackson Laboratories, Bar Harbor, ME), as described in detail previously^32^,^57^. In brief, hippocampal slices from 6-day old pups were cultured for 3 weeks on glass coverslips, mounted in a perfusion chamber and imaged by a home-built inverted STED microscope using a 100 × 1.4 NA oil immersion objective (PL APO 100, Leica) and a voxel size of 25 × 25 × 300 nm. We used pulsed lasers for excitation of YFP at 485 nm and for STED quenching at 595 nm, providing a lateral spatial resolution of approximately 50 nm. Spine morphology was analyzed using ImageJ on raw image sections. Spine neck widths were obtained as the full width at half maximum (FWHM) from Gaussian fits of line profiles through the neck region, each line being three pixels wide. Spine neck length was measured from the base of the spine head to the base of the dendrite, following the curvature of the neck. Only spines extending in the lateral plane were included in the morphological analysis and simulations. The displayed dendritic segments are surface renderings of z-stacks of 300 nm steps prepared using an ImageJ plugin^58^. Experimental procedures were in accordance with the French National Code of Ethics on Animal Experimentation and approved by the Committee of Ethics of Bordeaux (No. 50120202).

### Active compartmental models of dentate granule cells

For active simulations we employed an existing active model of hippocampal dentate gyrus granule cells with realistic biophysical properties previously published by Schmidt-Hieber *et al*.^54^. Passive properties were also taken from Schmidt-Hieber *et al*.^54^ to generate a passive structure for the insertion of voltage-dependent sodium and potassium channels^59^. Detailed 3D morphologies of granule cells (n = 8) were taken from Vuksic *et al*.^60^. The following parameters were used for explicit spines (Supplementary Table S5): head diameter 0.5 μm, head length 0.5 μm, neck diameter 0.18 μm and neck length 0.75 μm^54^. Implicit spines were modeled by scaling membrane resistance and capacitance of dendrites to incorporate the spine membrane surface area. Parameters used in our simulations were as follows: [Cl^−^]_i_ = 5 mM, [Cl^−^]_o_ = 133.5 mM, [HCO_3_^−^]_i_ = 16 mM, [HCO_3_^−^]_o_ = 26 mM, E_GABA_ = −68.63 mV, temperature 35 °C, P = 0.25, D_Cl_ = 2 μm^2^/ms; synapses: tau_1_ = 0.5 ms, tau_2_ = 6 ms, g_max_ = 1.5 nS, V_rest_ = −70 mV. GABAergic synapses with density of 0.5 synapse/μm were distributed along the dendrites in the outer molecular layer and the middle molecular layer.

### Statistical analysis

Statistical comparisons were made using paired Mann-Whitney test. P-values of less than 0.05 were considered significant. All values are expressed as mean ± SD.

## Additional Information

**How to cite this article**: Mohapatra, N. *et al*. Spines slow down dendritic chloride diffusion and affect short-term ionic plasticity of GABAergic inhibition. *Sci. Rep*. **6**, 23196; doi: 10.1038/srep23196 (2016).

## Supplementary Material

Supplementary Information

Supplementary Movie S7
