# Supplementary material for: Spines slow down dendritic chloride diffusion and affect short-term ionic plasticity of GABAergic inhibition
Source: Sci Rep. 2016 Mar 18;6:23196. doi: 10.1038/srep23196 (PMC4796789; doi:10.1038/srep23196)
Supplement: Supplementary Information [file srep23196-s1.pdf]

Spines slow down dendritic chloride diffusion and affect short-term ionic plasticity of GABAergic inhibition

Namrata Mohapatra, Jan Tønnesen, Andreas Vlachos, Thomas Kuner, Thomas Deller, U. Valentin Nägerl, Fidel Santamaria, Peter Jedlicka

Supplementary figures, tables and a movie

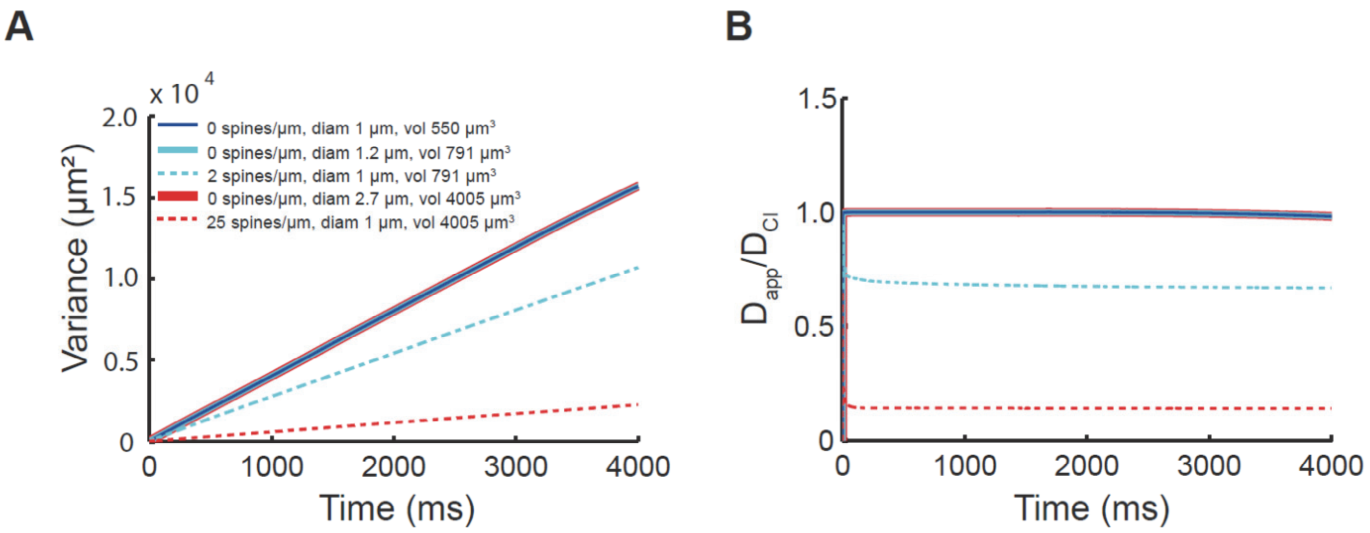

Supplementary figure S1: Increased diameter and volume of the dendrite does not slow down Cl^{-} diffusion

(**A**) The spatial variance of Cl^{-} concentration as a function of time did not change following the adjustment of the diameter in smooth dendrites. Diameters of smooth dendrites were increased to match the volume of spiny dendrites. The dotted lines represent sublinear time dependence of spatial variance indicating a slowdown of diffusion in spiny dendrites. (**B**) The instantaneous apparent diffusion coefficient (D_{app}) was computed from spatial variance and divided by the diffusion coefficient for Cl^{-} (D_{Cl}=2 um^2/ms).

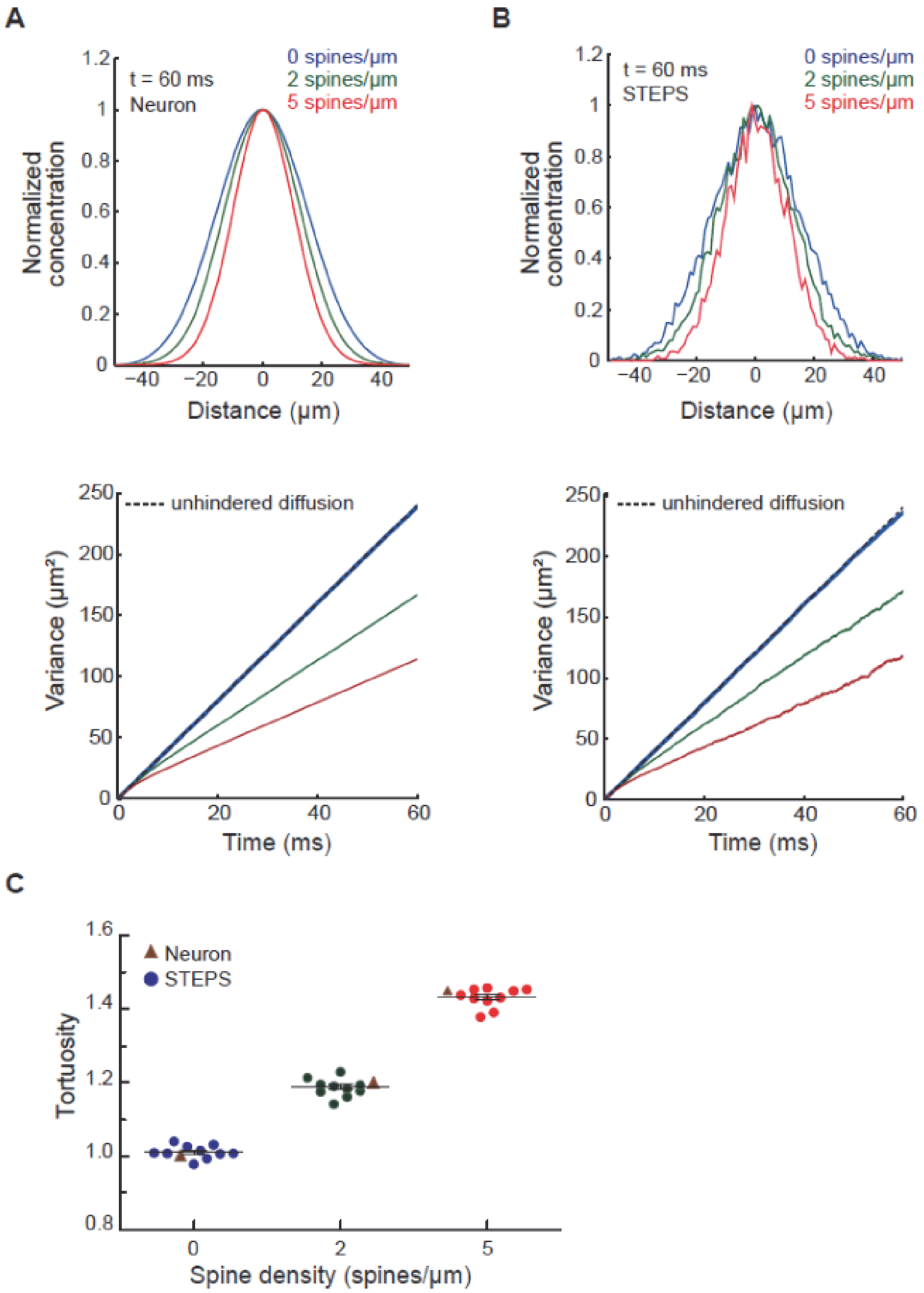

Supplementary figure S2: The slowdown of Cl^- diffusion caused by spines is present in both deterministic and stochastic simulations

(**A**) Cl^- diffusion in deterministic NEURON simulations. (**B**) Cl^- diffusion in stochastic STEPS simulations (10 iterations). Diffusion was triggered by an increase of Cl^- concentration at the center of the dendrite. Top in A, B: Normalized Cl^- concentration profiles at t = 60 ms in a dendritic cylinder of 100 µm length and 1 µm diameter. Bottom in A, B: The spatial variance of Cl^- concentration. The dotted line denotes the linear time dependence of the spatial variance in the case of free dendritic diffusion. Note altered time dependence of spatial variance in spiny dendrites indicating a slowdown of diffusion in deterministic as well as stochastic simulations. (**C**) The increase in tortuosity (λ) with increasing spine densities was similar in deterministic and in stochastic simulations.

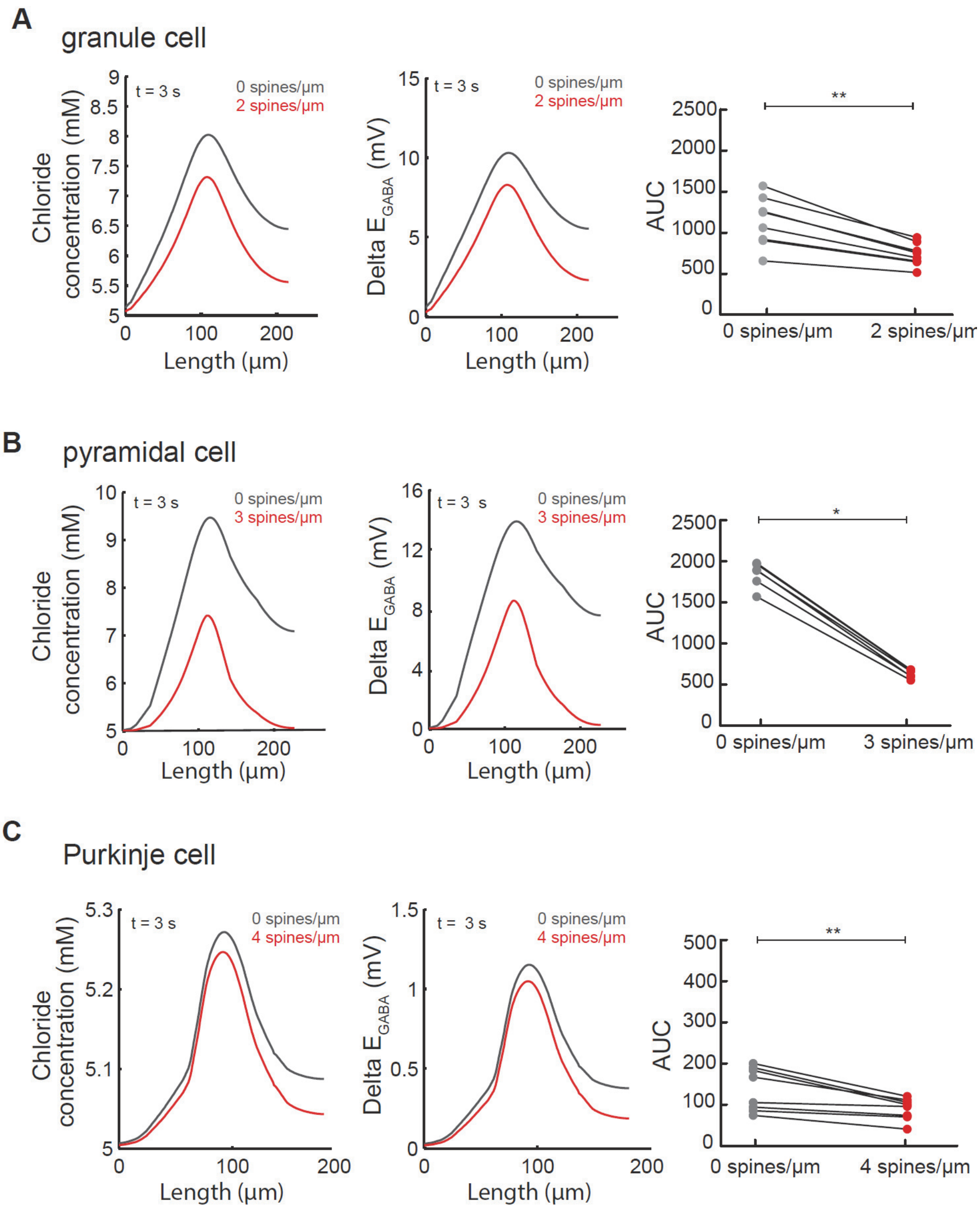

Supplementary figure S3: Spatial spread of short-term ionic plasticity in reconstructed dendritic trees with realistic branching

Left: Spatial profiles of activity-dependent E_{GABA} shift across the length of dendritic path which contained active GABAergic inputs (11 synapses, density 0.5/µm, rise time 0.5ms, decay time 6 ms, conductance 1 nS, duration of stochastic synaptic activity 3000 ms). Right: Quantification using the area under curve (AUC). (A-C) Results for dentate granule cells, CA1 pyramidal cells and Purkinje cells, respectively. Note that spines decrease activity-dependent ionic plasticity of E_{GABA}.

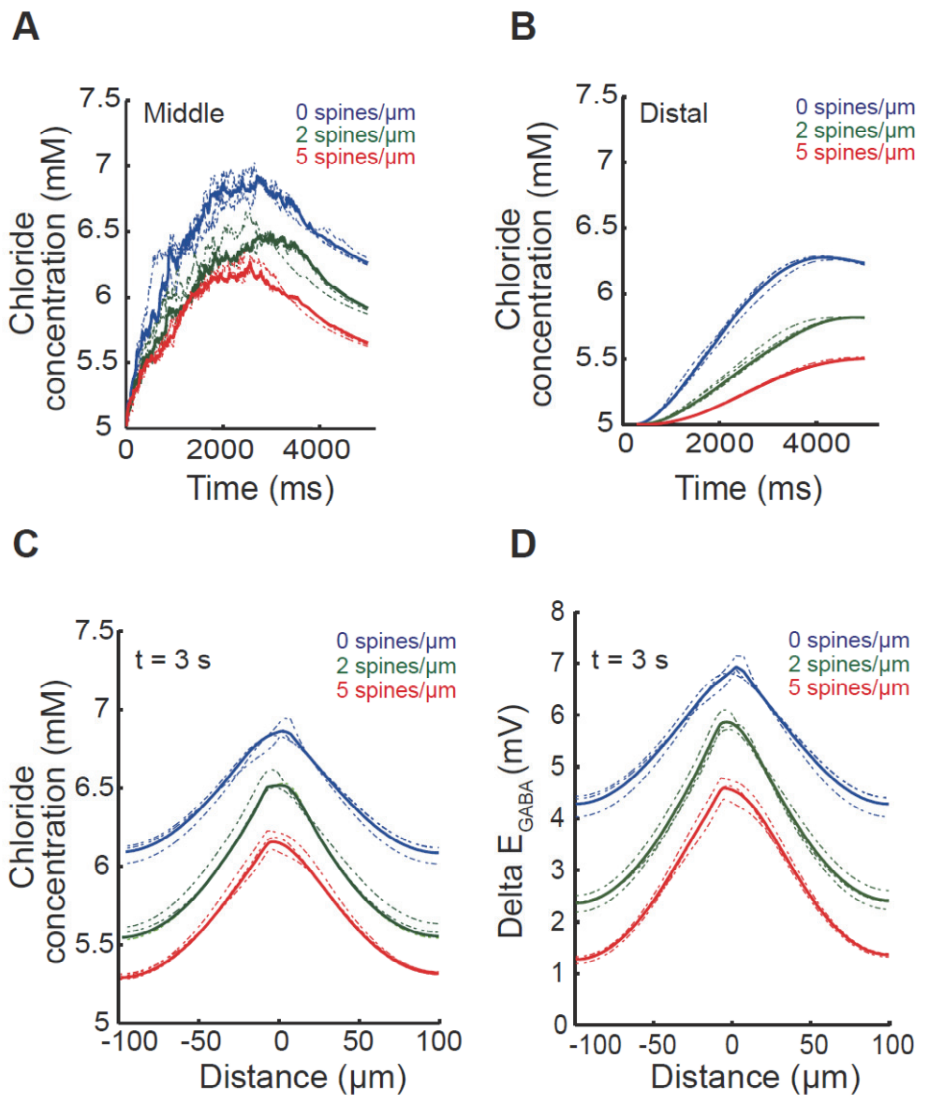

Supplementary figure S4: Spines affect short-term ionic plasticity induced by stochastic activation of GABAergic inputs

GABAergic synapses were inserted into a dendritic cylinder as in Fig. 5. **(A, B)** Changes of Cl^- concentration in the middle (A) and at the distal end (B) of the dendrite evoked by stochastic (Poisson) activation (10 Hz, 30 pulses, 3 iterations) of GABAergic inputs. **(C, D)** Spatial profile of Cl^- accumulation and E_{GABA} shift across the length of the dendrite recorded at the end of GABAergic activity. Note that, in the presence of spines, both homosynaptic as well as heterosynaptic ionic plasticity was reduced. Individual runs and their mean are displayed as dashed and solid lines, respectively.

Supplementary table S5: Morphological parameters of simulated dendritic spines

Figure	Spine Neck		Spine head	
	**Diameter**	**Length**	**Diameter**	**Length**
Figure 1*	0.20	1.25	0.60	0.55
Figure 2*	0.20	1.25	0.60	0.55
Figure 3 (cell1)**	0.17±0.05	0.60±0.50	0.72±0.18	0.56±0.22
Figure 3 (cell2)**	0.19±0.06	0.76±0.52	0.69±0.26	0.54±0.19
Figure 4	Heterogeneous spine parameters: Suppl. Table 2			
Figure 5*	0.20	1.25	0.60	0.55
Figure 6***	0.18	0.75	0.5	0.5
Figure S1*	0.20	1.25	0.60	0.55
Figure S2*	0.20	1.25	0.60	0.55
Figure S3	Heterogeneous spine parameters: Suppl. Table 2			
Figure S4*	0.20	1.25	0.60	0.55

Supplementary table S6: Morphological parameters of simulated spines used in reconstructed cells (Fig. 4 and S3)

	Mean	Variance	Range
**CA1-Pyramidal Cells***			
Neck Length	0.51	0.24	0.08-1.37
Neck Diameter	0.18	0.07	0.04-0.46
Head Length	0.52	0.37	0.15-1.89
Head Volume	0.18	0.09	0.005-0.51
**Purkinje Cells***			
Neck Length	0.68	0.30	0.12-2.18
Neck Diameter	0.18	0.05	0.09 – 0.31
Head Length	0.54	0.70	0.43-0.68
Head Volume	0.18	0.08	0.04-0.40
**Granule Cells******			
Neck Volume	0.0035	0.017	0.00007-0.180
Neck Length	0.26	0.17	0.03-0.94
Head Volume	0.038	0.04	0.003-0.225
Head Diameter	0.33	0.13	0.14-0.74

* Santamaria F, Wils S, De Schutter E, Augustine GJ. Anomalous Diffusion in Purkinje Cell Dendrites Caused by Spines. Neuron. 2006;52(4):635–48.

** Own data obtained by STED microscopy

*** Schmidt-Hieber C, Bischofberger J. Fast sodium channel gating supports localized and efficient axonal action potential initiation. J Neurosci. 2010;30(30):10233–42.

**** Trommald M, Hulleberg G. Dimensions and density of dendritic spines from rat dentate granule cells based on reconstructions from serial electron micrographs. J Comp Neurol 1997 377:15–28.

Supplementary movie S7: Simulated stochastic diffusion of Cl^- in a smooth dendrite and spiny dendrites. The video illustrates the diffusion after the release of Cl^- particles in a 1 μm center spot. In the absence of dendritic spines, Cl^- ions diffuse freely along the dendrite (top panel). Note that Cl^- ions enter spine necks and heads which leads to their decreased lateral movement inside spiny dendrites (second, third and fourth panel from the top: 2, 5 and 10 spines/μm, respectively). Simulations were run in STEPS.
